# Assessment of Levels of Natural Radioactivity in Sand Samples Collected from Ekalakala in Machakos County, Kenya

**DOI:** 10.1155/2020/7269840

**Published:** 2020-05-05

**Authors:** Lucia Nyiva Munyao, Daniel Kipngetich Ketui, Calford Otieno, Margaret Wairimu Chege

**Affiliations:** ^1^Department of Physics, Kisii University, P.O.Box. 408-40200, Kisii, Kenya; ^2^Department of Physics, Kenyatta University, P.O.Box. 443844, Nairobi, Kenya

## Abstract

Construction sand is naturally polluted with radionuclides of terrestrial origin. In this study, specific activities of ^238^U, ^232^Th, and ^40^K in 30 sand samples collected along the Ekalakala River, Machakos County, Kenya, were measured using a high-purity germanium *γ*-ray spectrometer. The specific activities ranged between 9.7 Bqkg^−1^ and 24.0 Bqkg^−1^, 11.5 Bqkg^−1^ and 26.2 Bqkg^−1^, and 820 Bqkg^−1^ and 1850 Bqkg^−1^ for ^238^U, ^232^Th, and ^40^K, respectively. While the mean specific activities for ^238^U and ^232^Th were less than half of the world average values of 33 Bqkg^−1^ and 45 Bqkg^−1^, respectively, the average specific activity of ^40^K was significant since it was three times the global mean value of 420 Bqkg^−1^. A calculated absorbed radiation dose rate for the sand varied between 46.8 nGyh^−1^ and 94.2 nGyh^−1^ with a mean of 68.5 ± 13.3 nGyh^−1^. This is not significantly different from the world average dose rate of 60 nGyh^−1^ for geological samples. The AEDR and H_ex_ had maximum values of 0.29 mSvy^−1^ and 0.52, respectively, both within the recommended limits of radiation exposure for members of the general public. Based on these results, the sand from Ekalakala River does not pose significant health implication to the sand harvesters as well as the inhabitants of the houses constructed using this sand.

## 1. Introduction

**Figure 1:**
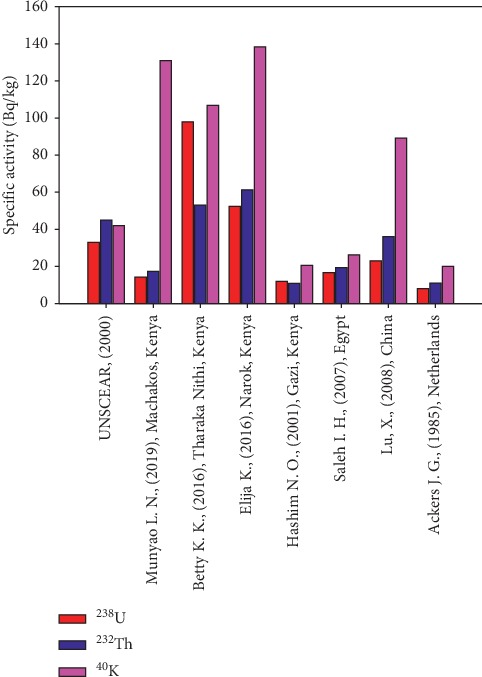
Comparison of the specific activity of ^238^U, ^232^Th, and ^40^K obtained in this study with values obtained in other parts of Kenya and the world. The specific activity of ^40^K has been scaled down by a factor of 10 for clear display of ^238^U and ^232^Th levels [5,10–15].

Sand mining is a well-known and booming economic activity in various regions of Kenya such as Machakos County [1]. This is as a result of the rapid economic expansion in most of the neighbouring cities and towns such as Nairobi, Thika, and Machakos, which has in turn resulted in an increased demand for the commodity in the construction industry. Sand is famously known as a construction material among other materials such as stones, gravel, and cement [2–4]. Just like any other type of geological materials, it naturally contains primordial radioactive nuclides ^238^U, ^232^Th, and ^40^K [5] which may not only contribute to external and internal radiation exposure to sand harvesters but also to the residents of the neighbouring towns. The risk posed by radiation exposure of terrestrial and extraterrestrial origin depends on, among other factors, the duration of exposure [6, 7]. Due to this, sand harvesters may be at a higher risk since they may be exposed to ionizing radiation both at work and in their homes. The risk also depends on the concentration of the primordial radionuclides [8] in the sand. Despite the continued harvesting and use of the construction sand from Ekalakala, there is no information about the radioactivity levels of the sand and possible health effects associated with continuous exposure of the sand harvesters as well as inhabitants of houses built using this sand. However, studies on construction sand obtained from other parts of Kenya have been carried out [5,9], and the values obtained are within the range of values obtained in this study. Moreover, comparison has been done on the values of ^238^U, ^232^Th, and ^40^K obtained in this study with other values obtained in Kenya [5,10,11] and different parts of the world [12–14] as shown in Figure 1. This study sought to fill the gap in determining the radiation exposure levels associated with construction sand from Ekalakala, by determining the specific activities of ^238^U, ^232^Th, and ^40^K in construction sand samples, evaluating the radiation absorbed dose rate as well as determining both the annual effective dose rate and the external hazard index. The results from this study seeks to provide knowledge and data on exposure levels associated with radionuclides in the construction sand and also avail the baseline information for future monitoring of the construction sand. They are as well vital in regulatory and advisory policy making for the public safety due to radiation exposure. Generally, this study acts as an eye opener to both the residents and local authorities to know that sand harvesting can attract more serious implications if not well monitored.

## 2. Materials and Methods

### 2.1. Study Area

**Figure 2:**
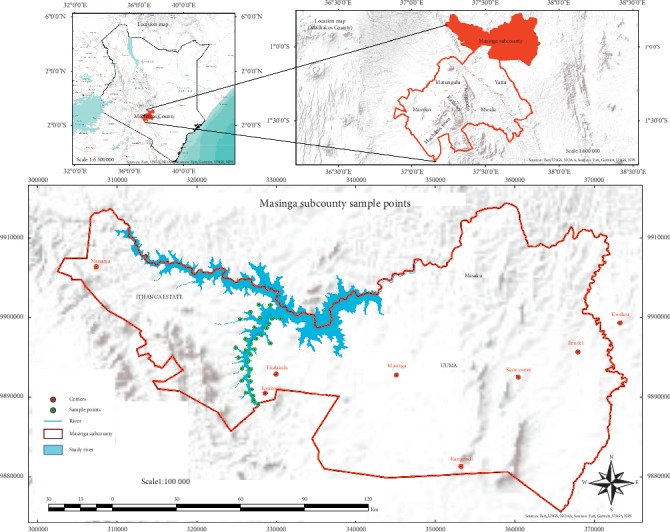
Location of sampling sites along Ekalakala River.

Machakos County is in the former eastern province of Kenya, it ranges between latitudes 0° 45ʹ South and 1°31ʹ South and stretches along longitudes 36°45ʹ East to 37°45ʹ East. Its altitude is 1000–1600 metres above sea level. It covers an area of 6,208 km^2^ and has a population of 1,098,584 with 264,500 [16] households. Sand samples were collected along Ekalakala River through the Isyukoni village eight kilometers from the Ekalakala market (Figure 2). Ekalakala River originates from the Aberdares forest and has several names from its origin. In Kiambu County, River Thika combines with River Sagana to form River Chania in Thika which in turn stretches to River Ianguni in Machakos County and then to Ekalakala River which pours its waters into Masinga dam.

### 2.2. Sample Collection and Preparation

A total of 30 sand samples each of mass 500 g were collected using a hand trowel into clean plastic containers. 15 of the samples were collected from depths of between 0 m and 0.3 m. Another set of 15 samples were collected directly beneath the first set at depths of between 0.3 m and 0.5 m. All samples were prepared for analysis at the Institute of Nuclear Science and Technology (INST), University of Nairobi, Kenya. In the laboratory, each sample was sieved so as to get rid of any unwanted materials including vegetation, stones, and debris. The samples were totally dried in an oven at 105°C for a day until a uniform weight was attained [4,17,18] so as to fully get rid of any water content. Dry sand was thereafter crushed and pulverized to uniformity, and then it was sieved using a 2 mm mesh sieve. 300 g of each sample was measured and put in standard gas tight plastic containers. The caps of these containers were carefully lined using an aluminium foil before sealing so as to ensure that ^222^Rn does not escape. Dimensions of the containers as well as the mass of the samples were similar to the IAEA reference used for efficiency calibration. All the containers were carefully labeled and stored in a cool dry place for a period of 30 days after which radioactivity was measured. This allowed radionuclides ^238^U and ^232^Th, radon and thoron, and short-lived radon and thoron progeny to attain secular equilibrium with each other [19,20].

## 3. Experimental Techniques

### 3.1. Radioactivity Measurement

For the detection of radioactivity in the sand samples, a high-purity germanium *γ*-ray detector, model number CPVD530–3018 SN 2489, was used. It was vertically mounted and stored in a 10 cm thick cylindrical lead shield which helped to lower the environmental background radiation. It was cooled using liquid nitrogen. The detector used is coaxial with a diameter of 57.4 mm, a length of 56.9 mm, and a volume of 144 cm^3^. It was connected to an uninterrupted power supply and operated at 3200 volts. It had 31.6% detection efficiency and a detector resolution (FWHM) of 1.8 keV at the 1.33 MeV of ^60^Co. Energy calibration of the detector was done using gamma energies 1330 keV and 1170 keV from ^60^Co and 60 keV from ^241^Am, while efficiency calibration was done using certified reference materials: RGU-1, RGTh-1, RGK-1, and IAEA soil 375. Samples were run for 36,000 seconds (10 hours), time considered adequate for counting sample radionuclide activity for each sample. ^232^Th specific activity was determined from the net intensity of radiation from energy photo peaks 238 keV of ^212^Pb and 911 keV of ^228^Ac. ^238^U was obtained from the 352 keV line of ^214^Pb and 609 keV of ^214^Bi. Finally, for ^40^K, it was obtained from its own gamma line of 1460 keV [21]. Specific activity (C) in Bqkg^−1^ was calculated using the following equation [22,23]:

(1) $$\begin{array}{c} C=\frac{A}{E_{\rho }\times \gamma \times T\times M_{S}}, \end{array}$$

where *A*  (count/s) is the net area under the photopeak for each sample, *E*_*ρ*_  is the detection efficiency at a specific gamma energy, *T* (s) is the counting time, *M*_*S*_ (kg) is the mass of samples, and *γ* is the gamma yield at a specific gamma energy. The lowest limits of detection in Bqkg^−1^ of the detector used were 4.1, 4.6, and 43.9 for ^238^U, ^232^Th, and ^40^K, respectively.

### 3.2. Absorbed Dose Rate Calculation

Dose rate is the dose of ionising radiation per unit time. The SI unit is gray per hour (Gyh^−1^). It was determined using the following formula [24]:

(2) $$\begin{array}{c} \dot{D}=0.427C_{\text{U}}+0.622C_{\text{Th}}+0.0432C_{\text{K}}, \end{array}$$

where *C*_U _, *C*_Th_ , and *C*_K_  are the specific activity in Bqkg^−1^ of ^238^U, ^232^Th, and ^40^ K, respectively, in the sand samples. 0.427, 0.662, and 0.043 are the dose conversion factors that convert the specific activity of ^238^U, ^232^Th, and ^40^ K into dose.

### 3.3. Annual Effective Dose Rate Calculation

AEDR is the equivalent biological effect representing the deposit of a joule of radiation energy per kilogram of a human body in a year. It was obtained by making use of the following equations [15,25–27]:

(3) $$\begin{array}{c} \text{indoor AEDR}=\dot{D}\times 8760\times 0.7\times 0.6\times 10^{-6}, \end{array}$$

(4) $$\begin{array}{c} \text{outdoor AEDR}=\dot{D}\times 8760\times 0.7\times 0.4\times 10^{-6}, \end{array}$$

where $\dot{D}$ is the dose rate, 8760 are the hours in a year, 0.7  SvGy^−1^ is the conversion coefficient that changes absorbed dose in the air to the effective dose, and 10^−6^  is a factor that converts nano into milli [22,28]. Finally, 0.6 and 0.4 are the estimated average indoor and outdoor occupancy factors in Kenya [5,7,29]. This takes into account the fact that Kenyans spent 40% of their time outside because the weather is favourable and 60% of their time is spent indoors. The SI unit of annual effective dose rate is mSvy^−1^.

### 3.4. External Hazard Index

The external hazard index resulting from exposure to gamma rays is determined by [30]

(5) $$\begin{array}{c} H_{\text{ex}}=\frac{C_{\text{U}}}{370}+\frac{C_{\text{Th}}}{259}+\frac{C_{\text{K}}}{4910}, \end{array}$$

where  *C*_U_, *C*_Th_, and *C*_K_ are the values of specific activity (BqKg^−1^) of ^238^U, ^232^Th, and ^40^K radionuclides, respectively. The value of this index should be lower than one. Values above unity make the hazard unacceptable to members of the general public [31].

## 4. Results and Discussion

**Figure 3:**
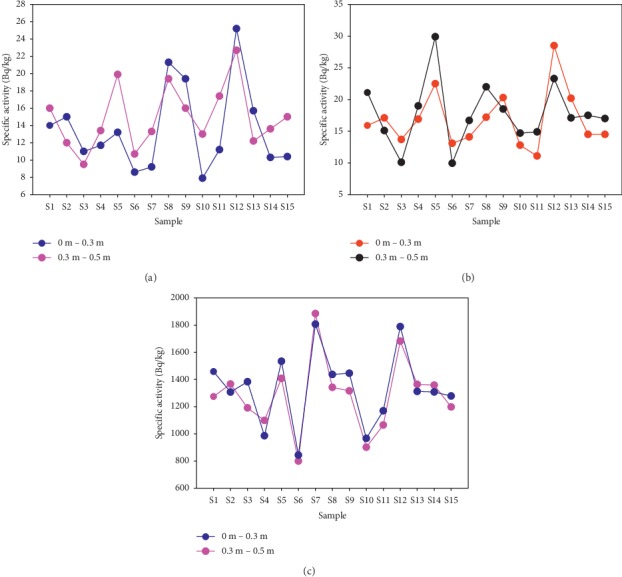
Comparison of the distribution of (a) ^238^U, (b) ^232^Th, and (c) ^40^K radionuclides for sand samples collected at a depth of 0 m and 0.3 m and those collected at a depth ranging from 0.3 m to 0.5 m.

Primordial radionuclides ^238^U, ^232^Th, and ^40^K were identified in the 30 sand samples. Values of standard deviation of the corresponding averages have been expressed as errors in all tables. The maximum value of ^238^U for samples collected between 0 m and 0.3 m was 25.2 ± 0.8 Bqkg^−1^, while the minimum value was 7.9 ± 3 Bqkg^−1^ with a mean of 13.6 ± 4.8 Bqkg^−1^ as shown column 2 in Table 1. For sand samples obtained between 0.3 m and 0.5 m, the specific activity ranged from 9.5 ± 0.8 Bqkg^−1^ to 22.7 ± 1 Bqkg^−1^ with an overall value of 14.9 ± 3.6 Bqkg^−1^ as indicated in Table 1 (column 3). Though the mean specific activity of ^238^U for sand samples collected at a depth of 0.3 m–0.5 m was higher than that collected at a depth of 0 m–0.3 m, there was an insignificant difference existing between the two sets of data. Further comparison of the specific activity of ^238^U with depth is displayed in Figure 3(a).

Specific activity of ^232^Th for sand samples collected at a depth of 0 m–0.3 m in Bqkg^−1^ ranged between 11.1 ± 3.1 and 28.5 ± 3.7 with an average of 16.8 ± 4.4 which is slightly lower than the average activity of the same radionuclide obtained for samples collected at a depth ranging between 0.3 m and 0.5 m as indicated in Table 2. The maximum specific activity of sand samples collected at a depth of 0.3 m–0.5 m in Bqkg^−1^ was 29.9 ± 1.4 with a minimum of 9.95 ± 2.1 and an average of 17.8 ± 4 as indicated in Table 2 (column 3). Distribution of ^232^Th at various sampling points is displayed in Figure 3(b). The difference existing between the specific activity of ^232^Th for sand samples obtained from a depth of 0 m–0.3 m and those collected at a depth of 0.3 m–0.5 m was found to be insignificant.

For ^40^K, the specific activity of samples collected between 0 m and 0.3 m ranged between 800 ± 60 Bqkg^−1^ and 1880 ± 30 Bqkg^−1^ with a mean value of 1300 ± 300 Bqkg^−1^ as indicated in Table 3 (column 2). The measured activity for sand samples collected at a depth of 0.3 m–0.5 m ranged between 840 ± 70 Bqkg^−1^ and 1800 ± 50 Bqkg^−1^ with a mean of 1300 ± 300 Bqkg^−1^ as indicated in Table 3 (column 3). The difference existing between the two sets of data is insignificant. The distribution of ^40^K at various sampling depths has been displayed in Figure 3(c).

**Figure 4:**
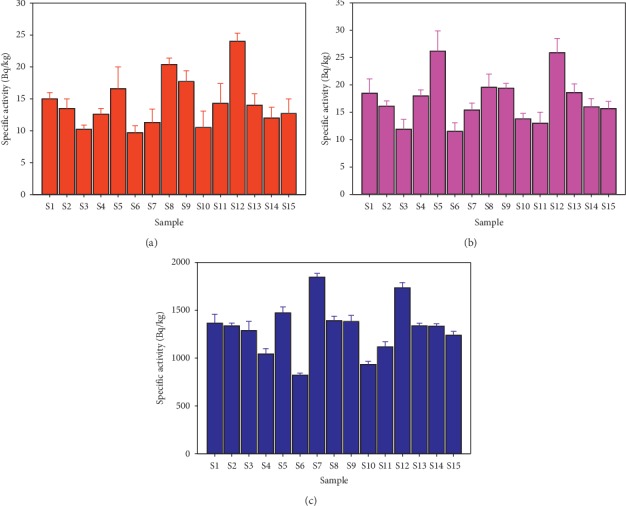
Distribution of (a) ^238^U, (b) ^232^Th, and (c) ^40^K in different sampling points.

The average specific activities in Bqkg^−1^ for the samples collected at the depths of 0 m–0.3 m and 0.3 m–0.5 m are shown in Table 4 and summarized in Figure 4. The overall mean activity for the data in this table was calculated to be 14.3 ± 3.8, 17.3 ± 4.2, and 1300 ± 300 for ^238^U, ^232^Th, and ^40^K, respectively. However, the specific activity of ^40^K obtained in this study is higher than the global mean value of 420Bqkg^−1^ [15].

This high value is attributed to the use of phosphate fertilizers for agriculture done on the upper parts of the river. Fertilizers are known to increase the concentration of ^40^K [25, 32]. It might as well be attributed to the fact that ^40^K is the most abundant radionuclide and is found in the earth's crust on an average of 2.6% [33]. Comparison of values of specific activity due to primordial radionuclides obtained in this study with values obtained nationally and internationally (Figure 1) indicates highest values of ^40^K activity followed by ^232^Th and ^238^U, that is, ^40^K > ^232^Th > ^238^U, except for [5, 11] whose data indicate slightly higher values of ^232^Th than those of ^238^U even though the values of ^40^K are highest in both cases.

**Figure 5:**
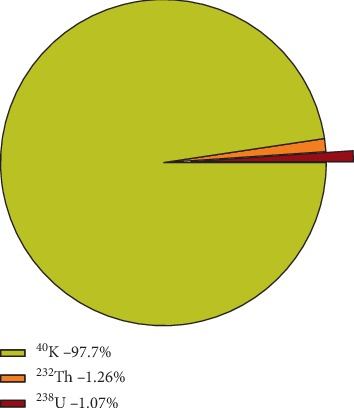
Percentage contribution of individual radionuclides to the average specific activity.

The percentage contribution of each radionuclide is indicated in Figure 5, which shows 97.7% of ^40^K, 1.26% of ^232^Th, and 1.07% of ^238^U which is in close agreement to values obtained in [34].

From Figure 1, the specific activity of ^40^K reported in this study is lower than values reported in Narok, Kenya [10], but higher than the global average value [15] and values reported in other parts of the world [5, 11–14]. ^238^U recorded in this study is lower than the worldwide mean [15] and values obtained in [5, 10, 13, 14] but higher than values reported in [11, 12].

Specific activity of ^232^Th obtained in the present study is lower than the worldwide average [15] as well as values recorded in [5, 10, 13, 14] but higher than values reported in [11, 12].

Correlation analysis performed between specific activities of ^238^U, ^232^Th, and ^40^K revealed that they all had a positive correlation with each other as shown in Table 5. This shows a strong degree of closeness among different radionuclides. It further implies that knowing the specific activity of one radionuclide can help in predicting the specific activity of the other radionuclide of interest in the study area.

**Figure 6:**
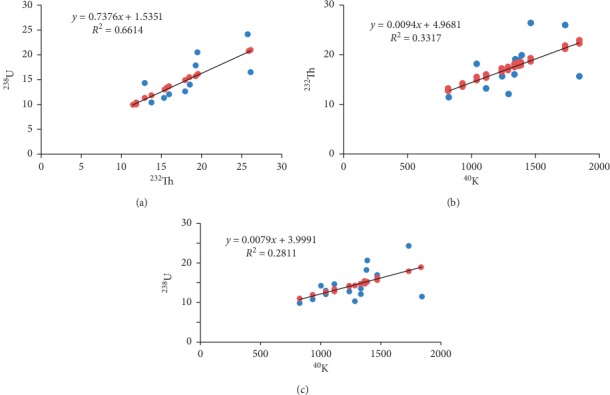
Regression plots showing the correlation between (a) ^238^U and ^232^Th, (b) ^232^Th and ^40^K, and (c) ^238^U versus ^40^K in sand samples analysed.

Regression plots showing the correlation between the three radionuclides are displayed in Figure 6. ^232^Th has a strong positive correlation with ^238^U (*r* = 0.81). However, a relatively strong positive correlation is evident between the specific activities of ^40^K and ^238^U (*r* = 0.53) and between the specific activities of ^40^K and ^232^Th (*r* = 0.58). This could possibly be due to the fact that radionuclides originate from the same rock formation.

**Figure 7:**
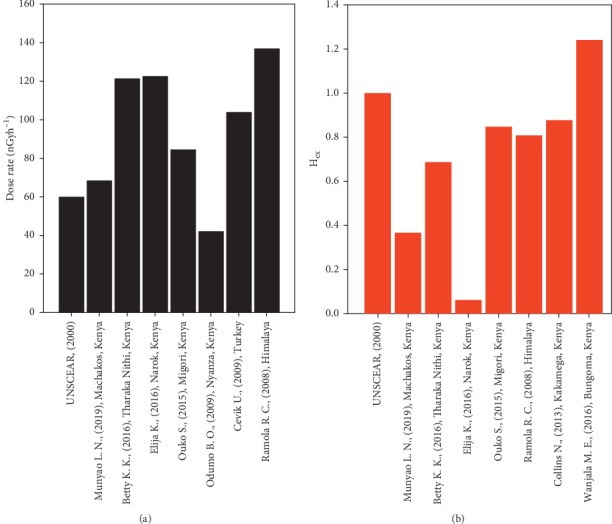
Comparison of the average (a) AEDR and (b) *H*_ex_ obtained in this study with world average values and values obtained in other parts of the world [5, 9, 10, 15, 33, 35–37].

The total absorbed dose rate (nGyh^−1^) due to terrestrial gamma radiation was calculated from the mean specific activity of ^238^U, ^232^Th, and ^40^K in the sand samples using equation (2). The dose rate ranged between 46.8 nGyh^−1^ and 94.2 nGyh^−1^ with a mean value of 68.5 ± 13.3 nGyh^−1^, as indicated in Table 6 (column 2). The values obtained have been compared to values obtained in different parts of the world (Figure 7(a)).

**Figure 8:**
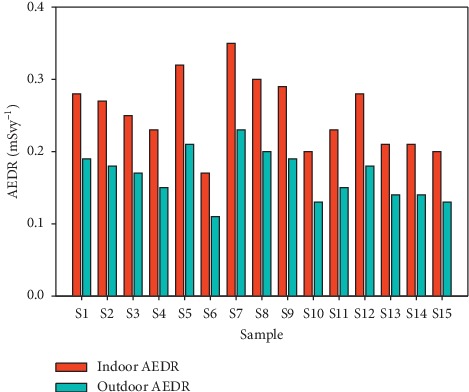
Comparison of the indoor and outdoor AEDR for all the sand samples.

The AEDR due to indoor and outdoor exposure was determined from equations (3) and (4). The mean indoor AEDR is 0.25 mSvy^−1^, while the average outdoor AEDR is 0.17 mSvy^−1^ as indicated in columns 2 and 3 in Table 7, respectively. The total AEDR varied from 0.29 mSvy^−1^ to 0.14 mSvy^−1^ with a mean of 0.23 ± 0.04 mSvy^−1^ as in column 4 in Table 7 and in Figure 8.

The average value obtained is lower than the recommended safety limit of 1 mSvy^−1^ for members of the general public. From a radiological point of view, this implies that the dose emitted from natural gamma does not pose any significant health implication to the sand harvesters as well as the inhabitants of the houses constructed using this sand. The ^238^U/^232^Th ratio is less than one as shown in Table 6 (column 4). This can be explained by the high solubility of uranium ions as compared to thorium ions which are slightly soluble.

The *H*_ex_ index, as calculated from equation (5), has a maximum value of 0.52, while the minimum value is 0.24 with a mean of 0.37, as indicated in column 3 in Table 6. These values have been compared to values obtained in Kenya and other parts of the world as displayed in Figure 7(b). Since the *H*_ex_ index is less than the global permissible limit of unity, the radiation hazard posed is negligible. This implies that the construction sand from Ekalakala may be considered safe for use by members of the general public.

## 5. Conclusion

The levels of natural radioactivity in sand samples collected from Ekalakala River, Machakos County, Kenya, have been assessed using an HPGe detector. The mean values of specific activity in Bqkg^−1^ are indicated in Table 4. The maximum specific activity of ^238^U and ^232^Th is 24.0 ± 1.3 Bqkg^−1^ and 26.2 ± 3.7 Bqkg^−1^, respectively, which is below the set values of 33 Bqkg^−1^ and 45 Bqkg^−1^, respectively. The maximum value of the specific activity of ^40^K reported is 1850 ± 40 Bqkg^−1^ which is higher than the worldwide mean value of 420 Bqkg^−1^. The average dose rate of 68.5 ± 13.3 nGyh^−1^ is slightly higher than the worldwide average value. The average indoor and outdoor AEDR was reported, and the values obtained were 0.25 ± 0.05 mSvy^−1^ and 0.17 ± 0.03 mSvy^−1^, respectively. The total AEDR due to indoor and outdoor exposure to gamma radiation is 0.21 mSvy^−1^, which is lower than the recommended safety limit of 1 mSvy^−1^. The H_ex_ index was also reported. This value was below the set limit of unity. The construction sand from Ekalakala River poses an insignificant health risk to members of the general public. These results can be of great use by the relevant governmental organizations in coming up with suitable policies on radiation protection and control. It can as well be used as reference data in future to monitor possible radioactivity pollution from the construction sand from Ekalakala River.

## Figures and Tables

**Table 1:** Specific activity in Bqkg^−1^ for ^238^U in sand samples collected at depths 0 m–0.3 m and 0.3 m–0.5 m from Ekalakala River.

Sample	Specific activity (Bqkg^−1^)	
	0 m–0.3 m	0.3 m–0.5 m
1	14 ± 1.4	16 ± 5.0
2	15 ± 1.5	12 ± 0.6
3	11.0 ± 0.6	9.5 ± 0.8
4	11.7 ± 0.1	13.4 ± 2.1
5	13.2 ± 0.1	19.9 ± 0.8
6	8.6 ± 1.0	10.7 ± 0.3
7	9.2 ± 0.8	13.3 ± 2.0
8	21.3 ± 1.3	19.4 ± 1.1
9	19.4 ± 0.6	16 ± 3.3
10	7.9 ± 3.0	13 ± 0.5
11	11.2 ± 0.2	17.4 ± 0.1
12	25.2 ± 0.8	22.7 ± 1.0
13	15.7 ± 0.3	12.2 ± 0.2
14	10.3 ± 0.8	13.6 ± 0.4
15	10.4 ± 1.1	15 ± 1.2

Minimum	**7.9** **±** **3.0**	**9.5** **±** **0.8**
Maximum	**25.2** **±** **0.8**	**22.7** **±** **1.0**
Mean	**13.6** **±** **4.8**	**14.9** **±** **3.6**

**Table 2:** Specific activity of ^232^Th present in sand samples collected at depths of 0 m–0.3 m and 0.3 m–0.5 m from Ekalakala River.

Sample	Specific activity (Bqkg^−1^)	
	0 m–0.3 m	0.3 m–0.5 m
1	15.9 ± 1.7	21.1 ± 2.8
2	17.1 ± 1.1	15.1 ± 0.9
3	13.7 ± 0.8	10.1 ± 0.5
4	16.9 ± 2.1	19.0 ± 2.2
5	25.5 ± 2.3	29.9 ± 1.4
6	13.1 ± 2.5	9.95 ± 2.1
7	14.1 ± 4.1	16.7 ± 4.4
8	17.2 ± 2.7	22.0 ± 2.4
9	20.3 ± 0.3	18.5 ± 3.8
10	12.8 ± 2.4	14.7 ± 1.3
11	11.1 ± 3.1	14.9 ± 0.9
12	28.5 ± 3.7	23.3 ± 5.5
13	20.2 ± 4.8	17.1 ± 2.9
14	14.5 ± 2.4	17.5 ± 0.4
15	14.5 ± 1.9	17.0 ± 1.1

Minimum	**11.1** **±** **3.1**	**9.95** **±** **2.1**
Maximum	**28.5** **±** **3.7**	**29.9** **±** **1.4**
Mean	**16.8** **±** **4.4**	**17.8** **±** **4.9**

**Table 3:** Specific activity of the ^40^K radionuclide in sand samples collected at depths 0 m–0.3 m and 0.3 m–0.4 m from Ekalakala River.

Sample	Specific activity (Bqkg^−1^)	
	0 m–0.3 m	0.3 m–0.5 m
1	1270 ± 70	1460 ± 60
2	1370 ± 50	1300 ± 40
3	1190 ± 40	1380 ± 70
4	1100 ± 40	990 ± 50
5	1410 ± 60	1500 ± 70
6	800 ± 60	840 ± 70
7	1880 ± 30	1800 ± 50
8	1340 ± 80	1440 ± 40
9	1320 ± 30	1450 ± 50
10	900 ± 60	970 ± 60
11	1060 ± 40	1170 ± 50
12	1680 ± 60	1790 ± 70
13	1360 ± 50	1310 ± 60
14	1360 ± 30	1310 ± 70
15	1200 ± 60	1280 ± 70

Minimum	**800** **±** **60**	**840** **±** **70**
Maximum	**1880** **±** **30**	**1800** **±** **50**
Mean	**1300** **±** **300**	**1300** **±** **300**

**Table 4:** Average specific activity of ^238^U, ^232^Th, and ^40^K calculated from sand samples collected at depths of 0 m–0.3 m and 0.3 m–0.5 m from Ekalakala River.

Sample	Specific activity (Bqkg^−1^)		
	^238^U	^232^Th	^40^K
1	15.0 ± 1.0	18.5 ± 2.6	1370 ± 90
2	13.5 ± 1.5	16.1 ± 1.0	1340 ± 30
3	10.2 ± 0.7	11.9 ± 1.8	1290 ± 100
4	12.6 ± 0.9	18.0 ± 1.1	1040 ± 60
5	16.6 ± 3.4	26.2 ± 3.7	1470 ± 60
6	9.7 ± 1.1	11.5 ± 1.6	820 ± 20
7	11.3 ± 2.1	15.4 ± 1.3	1850 ± 40
8	20.4 ± 1.0	19.6 ± 2.4	1390 ± 50
9	17.7 ± 1.7	19.4 ± 0.9	1380 ± 70
10	10.5 ± 2.6	13.8 ± 1.0	930 ± 30
11	14.3 ± 3.1	13.0 ± 2.0	1120 ± 50
12	24.0 ± 1.3	25.9 ± 2.6	1740 ± 50
13	14.0 ± 1.8	18.6 ± 1.6	1340 ± 30
14	12.0 ± 1.7	16.0 ± 1.5	1330 ± 30
15	12.7 ± 2.3	15.7 ± 1.3	1240 ± 40

Range	**9.7–24.0**	**11.5–26.2**	**820–1850**
Mean	**14.3** **±** **3.8**	**17.3** **±** **4.2**	**1300** **±** **300**
World mean	**33**	**45**	**420**

**Table 5:** Correlation matrix table for the specific activities of ^238^U, ^232^Th, and ^40^K.

	^238^U	^232^Th	^40^K
^238^U	1		
^232^Th	0.8133	1	
^40^K	0.5302	0.5760	1

**Table 6:** Dose rate (nGyh^−1^), external hazard index, and ^238^U/^232^Th ratio for sand samples collected from Ekalakala River.

Sample	Dose (nGyh^−1^)	H_ex_	^238^U/^232^Th
1	76.9	0.39	0.81
2	73.5	0.37	0.84
3	67.4	0.34	0.86
4	61.6	0.32	0.7
5	86.9	0.45	0.63
6	46.8	0.24	0.84
7	94.2	0.47	0.73
8	80.9	0.41	1.04
9	79.3	0.40	0.91
10	53.4	0.27	0.76
11	62.4	0.32	1.1
12	75.0	0.52	0.93
13	57.8	0.38	0.75
14	57.6	0.37	0.75
15	53.5	0.35	0.81

Maximum	**94.2**	**0.52**	**1.1**
Minimum	**46.8**	**0.24**	**0.63**
Average	**68.5** **±** **13.3**	**0.37** **±** **0.07**	**0.83** **±** **0.12**

**Table 7:** Indoor, outdoor, and average annual effective dose rate (mSvy^−1^) for sand samples collected at Ekalakala River.

Sample	Indoor AEDR (mSvy^−1^)	Outdoor AEDR (mSvy^−1^)	Total AEDR (mSvy^−1^)
1	0.28	0.19	0.24
2	0.27	0.18	0.23
3	0.25	0.17	0.21
4	0.23	0.15	0.19
5	0.32	0.21	0.27
6	0.17	0.11	0.14
7	0.35	0.23	0.29
8	0.30	0.20	0.25
9	0.29	0.19	0.24
10	0.2	0.13	0.16
11	0.23	0.15	0.19
12	0.28	0.18	0.23
13	0.21	0.14	0.18
14	0.21	0.14	0.18
15	0.20	0.13	0.16

Maximum	**0.35**	**0.23**	**0.29**
Minimum	**0.17**	**0.11**	**0.14**
Average	**0.25** **±** **0.05**	**0.17** **±** **0.03**	**0.21** **±** **0.04**

## Data Availability

The raw data used to calculate the specific activity of ^238^U, ^232^Th, and ^40^K in this research have been deposited in the Mendeley data repository at http://doi.org/10.17632/87c9ysn8v6.1.
